# Ultramicronized *N*-palmitoylethanolamine Contributes to Morphine Efficacy Against Neuropathic Pain: Implication of Mast Cells and Glia

**DOI:** 10.2174/1570159X21666221128091453

**Published:** 2023-04-27

**Authors:** Laura Micheli, Elena Lucarini, Stefania Nobili, Gianluca Bartolucci, Marco Pallecchi, Alessandra Toti, Valentina Ferrara, Clara Ciampi, Carla Ghelardini, Lorenzo Di Cesare Mannelli

**Affiliations:** 1 Department of Neuroscience, Psychology, Drug Research and Child Health - NEUROFARBA - Pharmacology and Toxicology Section, University of Florence, Florence, Italy;; 2 Department of Neuroscience, Psychology, Drug Research and Child Health - NEUROFARBA - Pharmaceutical and Nutraceutical Sciences Section, University of Florence, Florence, 50019, Italy

**Keywords:** Ultramicronized *N*-palmitoylethanolamine (PEA), morphine, opioid, mast cell, glia, histamine, neuropathic pain

## Abstract

**Background:**

In the current management of neuropathic pain, in addition to antidepressants and anticonvulsants, the use of opioids is wide, despite their related and well-known issues.

**Objective:**

* N*-palmitoylethanolamine (PEA), a natural fatty-acid ethanolamide whose anti-inflammatory, neuroprotective, immune-modulating and anti-hyperalgesic activities are known, represents a promising candidate to modulate and/or potentiate the action of opioids.

**Methods:**

This study was designed to evaluate if the preemptive and morphine concomitant administration of ultramicronized PEA, according to fixed or increasing doses of both compounds, delays the onset of morphine tolerance and improves its analgesic efficacy in the chronic constriction injury (CCI) model of neuropathic pain in rats.

**Results:**

Behavioral experiments showed that the preemptive and co-administration of ultramicronized PEA significantly decreased the effective dose of morphine and delayed the onset of morphine tolerance. The activation of spinal microglia and astrocytes, commonly occurring both on opioid treatment and neuropathic pain, was investigated through GFAP and Iba-1 immunofluorescence. Both biomarkers were found to be increased in CCI untreated or morphine treated animals in a PEA-sensitive manner. The increased density of endoneural mast cells within the sciatic nerve of morphine-treated and untreated CCI rats was significantly reduced by ultramicronized PEA. The decrease of mast cell degranulation, evaluated in terms of reduced plasma levels of histamine and N-methyl-histamine metabolite, was mainly observed at intermediate-high doses of ultramicronized PEA, with or without morphine.

**Conclusion:**

Overall, these results show that the administration of ultramicronized PEA in CCI rats according to the study design fully fulfilled the hypotheses of this study.

## 1 INTRODUCTION

According to the International Association for the Study of Pain (IASP), neuropathic pain is the direct consequence of a lesion or disease of the somatosensory system [1]. It may depend on several causes (*e.g*., postherpetic neuralgia, diabetic or HIV-related neuropathy, neuropathic cancer-related pain, radiculopathy) and may strongly affect the patient quality of life [2]. Based on epidemiological data, up to 10% of the general population suffers from neuropathic pain [3]. The pharmacological treatment is mainly represented by multimodal analgesia. Since neuropathic pain does not usually respond to nonsteroidal anti-inflammatory drugs (NSAIDs) and acetaminophen, drugs most widely used, frequently as a combination, are antidepressants such as tricyclic antidepressants, serotonin-noradrenaline reuptake inhibitors (SNRI), anticonvulsants such as pregabalin and strong opioids [4-6]. In addition, in the clinical routine, opioids may also be recommended to patients for whom a definitive diagnosis cannot be performed (*i.e*., in the presence of “possible” neuropathic pain) [3]. Thus, although the use of strong opioids is generally associated with well-known side effects (*i.e*. induction of tolerance) [4, 7], their use remains wide. Therefore, the identification of substances able to modulate and/or potentiate the action of opioids by lowering their doses and increasing their analgesic effects represents a fundamental goal. *N*-palmitoylethanolamine (PEA) is a natural fatty-acid ethanolamide that belongs to the autacoid local injury antagonist amides (ALIAmides) family. The anti-inflammatory activity of PEA from egg yolk was observed for the first time at the end of the 1950s [8]. Thanks to the renewed interest in the study on autacoids of the Nobel Laureate Rita Levi Montalcini and her group [9], several properties of physiological PEA were then identified, including neuroprotective, immunomodulating and anti-hyperalgesic activities [10-17].

In particular, our group has previously shown that (i) the concomitant subcutaneous administration of micronized PEA (in its high bioavailable form (*i.e*., ultramicronized PEA)) with intraperitoneal morphine, successfully delayed the onset of morphine tolerance [10] and (ii) the preemptive and concomitant oral administration of ultramicronized PEA increased the acute antinociceptive efficacy of subcutaneously administered morphine and delayed the development of morphine tolerance in healthy rats [13]. The maintenance of adequate PEA levels through the preemptive PEA treatment strategy is determinant due to the observed reduced levels of endogenous PEA in the spinal cord after 3 days from CCI of the sciatic nerve [18]. More recently, by inhibiting one of the major hydrolyzing enzymes of PEA, *i.e*., N-acylethanolamine acid amidase (NAAA), we further highlighted the key role of endogenous PEA in improving the pharmacological profile of morphine [19]. However, all the aforementioned studies were performed in healthy animals and not in neuropathic animal models. Here, we wanted to explore whether the preemptive and concomitant administration of ultramicronized PEA with morphine delays the onset of morphine tolerance and potentiates morphine analgesia in chronic constriction injury (CCI)-induced neuropathic pain in rats. Moreover, the role of non-neuronal cells, strongly involved in neuropathic pain and opioid tolerance (*i.e*., glia and mast cells), as well as PEA mechanisms of action, were also investigated.

## 2 MATERIALS AND METHODS

### 2.1 Animals

For all the experiments described below, male Sprague-Dawley rats (Envigo, Varese, Italy) weighing approximately 250-300 g were used. Animals were housed in CeSAL (Centro Stabulazione Animali da Laboratorio, University of Florence) and allowed to acclimatize for at least one week after their arrival. Rats were housed as follows: four per cage (size 26 × 41 cm), kept at 23 ± 1°C with a 12 h light/dark cycle (light from 7 a.m) and were fed a standard laboratory diet and tap water ad libitum. All animal manipulations were carried out according to the Directive 2010/63/EU of the European parliament and of the European Union council (22 September 2010) on the protection of animals used for scientific purposes. The ethical policy of the University of Florence complies with the Guide for the Care and Use of Laboratory Animals of the US National Institutes of Health (NIH Publication No. 85-23, revised 1996; University of Florence assurance number: A5278-01). Formal approval to conduct the experiments herein described was obtained from the Animal Subjects Review Board of the University of Florence (OPBA, CESAL, Council for Animal Wellness). Experiments involving animals have been reported according to ARRIVE guidelines [20]. All efforts were made to minimize animal suffering and to reduce the number of animals used. A total of 90 animals were used, 10 rats for each experimental group.

### 2.2 Neuropathic Pain Induction by Chronic Constriction Injury (CCI)

Neuropathy was induced on day -8 according to the procedure described by Bennett and Xie [21]. Briefly, rats were anaesthetized with 2% isoflurane. Under aseptic conditions, the right (ipsilateral) common sciatic nerve was exposed at the level of the middle thigh by blunt dissection. Proximal to the trifurcation, the nerve was carefully freed from the surrounding connective tissue, and four chromic catgut ligatures (4-0, Ethicon, Norderstedt, Germany) were tied loosely around the nerve with about a 1 mm spacing between ligatures. After hemostasis was confirmed, the incision was closed in layers. The animals were allowed to recover from surgery and then housed one per cage with free access to water and standard laboratory chow. The Control group of animals was subjected to sham surgery in which the sciatic nerve was only exposed but not ligated.

### 2.3 Treatments

Ultramicronized PEA (gently provided by Epitech Group, Padua, Italy and hereafter referred to as PEA) was suspended in 1% carboxymethylcellulose (CMC) and daily administered by oral gavage (p.o) (in the evening from day -8 to day 0 (preemptive treatment) and from day 1 to the end of the experiments (*i.e*. ≥ 30 mg kg^-1^, according to the treatment group). As detailed below, rats from two groups also received a co-administration of increasing doses of PEA together with morphine. Morphine (S.A.L.A.R.S., Como, Italy) was dissolved in saline solution and daily subcutaneously (s.c.) administered starting from day 1 until the end of the experiments. The dose of morphine was different depending on the experiment, as detailed below and in Fig. (**1**). The volume of morphine and PEA administered was calculated on the animal’s body weight using the ratio of 1 kg/10 mL. Experiments were conducted and set up to evaluate if PEA:

1) Delays the onset of morphine tolerance in CCI rats (animals were divided into different groups and treated as follow):

- Group A: sham + vehicles (1% CMC p.o. from day -8 to day 15 and saline s.c. from day 1 to day 15).
- Group B: CCI + vehicles (1% CMC p.o. from day -8 to day 15 and saline s.c. from day 1 to day 15).
- Group C: CCI + morphine 10 mg kg^-1^ (from day 1 to day 15) + vehicle (1% CMC p.o from day -8 to day 15).
- Group D: CCI + preemptive PEA 30 mg kg^-1^ (from day -8 to day 15) + morphine 10 mg kg^-1^ (from day 1 to day 15).

2) Potentiates morphine analgesia in CCI rats and 3) reduces CCI-induced hyperalgesia (animals were divided into different groups and treated as follow):

- Group E: sham + vehicles (1% CMC p.o. from day -8 to day 23 and saline s.c. from day 1 to day 23).
- Group F: CCI + vehicles (1% CMC p.o. from day -8 to day 23 and saline s.c. from day 1 to day 23).
- Group G: CCI + increasing morphine 5-35 mg kg^-1^ (from day 1 to day 23) + vehicle (1% CMC p.o; from day -8 to day 23).
- Group H: CCI + preemptive PEA 30 mg kg^-1^ (from day -8 to day 23) + increasing morphine 5-7 mg kg^-1^ (from day 1 to day 23) + increasing acute PEA 30-60 mg kg^-1^ (from day 8 to day 23).
- Group I: CCI + preemptive PEA 30 mg kg^-1^ (from day -8 to day 4) and 60 mg kg^-1^ (days 5-23) + increasing acute PEA 30-120 mg kg^-1^ (from day 3 to day 23).

### 2.4 Paw-pressure Test

The nociceptive threshold of rats was determined with an analgesimeter (Ugo Basile, Varese, Italy), according to the method as described in Leighton *et al*. [22]. A constantly increasing pressure was applied to a small area of the dorsal surface of the hind paw using a blunt conical probe by a mechanical device. Mechanical pressure was increased until vocalization or a withdrawal reflex occurred while rats were lightly restrained. Vocalization or withdrawal reflex thresholds were expressed in grams. For analgesia measures, mechanical pressure application was stopped at 200 g. Behavioral measurements were performed before and 30 min after the morphine injection. Sham animals received an equal volume of vehicles.

### 2.5 Von Frey Test

The mechanical allodynia was measured with an electronic Von Frey hair unit (Ugo Basile, Varese, Italy) as described in previous studies [23, 24]. Briefly, animals were placed in 20 cm × 20 cm Plexiglas boxes equipped with a metallic mesh floor, 20 cm above the bench. Animals were allowed to get used to the environment for 10 min before the test. The withdrawal threshold was evaluated by applying forces ranging from 0 to 50 g with a 0.2 g accuracy. Punctuate stimulus was delivered to the mid-plantar area of each posterior paw from below the mesh floor through a plastic tip and the withdrawal threshold was automatically displayed on the screen. The paw sensitivity threshold was defined as the minimum force required to obtain a robust and immediate withdrawal reflex of the paw. Measurements were performed on the posterior paw. Voluntary movements associated with locomotion were not considered as a withdrawal response. Measurements were repeated 5 times and the final value was obtained by averaging the 5 measurements. Behavioral measurements were performed before and 30 min after the morphine injection. Sham animals received an equal volume of vehicles.

### 2.6 Incapacitance Test

Spontaneous pain was evaluated by an incapacitance apparatus (Linton Instrumentation, Norfolk, UK) to record variations in the postural equilibrium [25, 26]. Rats were trained to stand on their hind paws in a box that was located above the incapacitance apparatus. This allows performing an accurate measure of the weight that the rat applies on each hind limb. The data represent the mean of the five following measurements for each animal. The difference between the weight applied to the limb contralateral to the injury and the weight applied to the ipsilateral one (∆ weight) was considered. Behavioral measurements were performed before and 30 min after the morphine injection. Sham animals received an equal volume of vehicles.

### 2.7 Histological Studies for Mast Cells Analysis

At the end of the behavioral experiments (day 15 or 23), animals were sacrificed by decapitation. The sciatic nerve, dorsal root ganglions (DRGs), thoracic spinal cord and meninges from the spinal cord were dissected and stored in 4% paraformaldehyde overnight. Tissues were then washed in water, dehydrated through an increasing scale of alcohols, cleared in xylene and embedded in paraffin. Sections of 5 µm were obtained from each paraffin-embedded tissue. Mast cells were detected by histochemical GIEMSA staining (Sigma-Aldrich, Italy). Digitalized images were collected at 10x magnification by a Leica DMRB light microscope, equipped with a DFC480 digital camera (Leica Microsystems, UK), and analyzed using ImageJ software. Two blind investigators independently evaluated the cellular density (cell number/respective arbitrary field). The reported values are the means ± S.E.M of the measurements of individual animals. At least five independent arbitrary optic fields collected from each tissue of each animal were analyzed.

### 2.8 Immunohistochemistry of Spinal Cord

On the day of sacrifice, the rat lumbar spinal cord (L4-L5) was removed and placed in ice-cooled 4% paraformaldehyde (PFA) in 0.1 M phosphate-buffered saline (PBS). The spinal cord was transferred to 30% sucrose (w/v) in 0.1 M PBS for 24 h for cryoprotection and then embedded in the optimal cutting temperature (OCT) compound using dry-ice-cooled hexane. Formalin fixed cryostat sections of the lumbar spinal cord (5 μm) were incubated for 1 h in blocking solution (Bio-Optica; Italy) at room temperature, and thereafter sections were incubated for 24 h at 4°C in PBST containing primary antisera and 5% normal donkey serum. The primary antibody was directed against Iba-1 (rabbit antiserum, 1:500; Wako Chemicals, USA; [27]) for microglial staining and against the glial fibrillary acidic protein (GFAP; rabbit antiserum, 1:500; Dako, USA; [28]) for astrocyte staining. After rinsing in PBST, sections were incubated in donkey anti-rabbit IgG secondary antibody labelled with Alexa Fluor 488 (1:1000, Invitrogen, USA) at room temperature for 1 h. For all the immunohistochemical studies, negative control sections (no exposure to the primary antisera) were processed concurrently with the other sections. An optical density value for the ipsilateral and contralateral dorsal horns in each rat was obtained.

### 2.9 Quantitative Analyses of Iba-1 and GFAP Immunofluorescence

Images were acquired using a motorized Leica DM6000B microscope equipped with a DFC350FX camera. Quantitative analysis of GFAP and Iba-1 positive cells was performed by collecting at least three independent fields through a 20 × 0.5 NA objective. Quantification of GFAP signal in immunostained sections was also performed using FIJI software by automatic thresholding images with the aid of the “Moments” algorithm, which delivered the most consistent pattern recognition across all acquired images.

### 2.10 Histamine and N-methyl-histamine Determination in Rat Plasma Samples

The concentration of histamine and N-methyl-histamine in rat plasma samples was measured by two dimensional high performance liquid chromatography (2D-HPLC) coupled with a tandem mass spectrometry system (MS/MS). The proposed method takes advantage of the internal standard approach by using an isotopologue (isotopic dilution) of the analyte (2[H^4^] Histamine), that was added to the samples and distinguished from the analytes only during the detection. The isotopic dilution 2D-HPLC-MS/MS analyses were performed with a triple quadrupole mass spectrometer (QqQ) equipped with an electrospray source (ESI) in positive ion acquisition mode. The solvents for the 2D-HPLC pumps were: ultrapure water: CH_3_CN 9:1 solution added with 5 mM HCOOH and 15 mM HCOONH_4_ (Solvent A); ultrapure water:CH_3_CN 1:9 solution added 15 mM HCOOH and 5 mM HCOONH_4_ (Solvent B); Solvent C (used for sample loading): ultrapure water:CH_3_CN 9:1 solution added with 17.5 mM HCOOH and 2.5 mM HCOONH_4_.

The analytical column used for the analytes assay was the SeQuant^®^ ZIC-HILIC 50 x 2.1 mm, 3.5 µm, 100 Å, while the loading column was the SeQuant^®^ ZIC-HILIC Guard 20 x 2.1mm. The rat plasma samples were diluted 1:10 with ultrapure water, and 20 μL of the final solution was injected in the 2D-HPLC-MS/MS system and analyzed following the method conditions reported in supporting information (**S1**) for a total analysis time of 25 minutes per sample.

### 2.11 Statistical Analysis

Trained observers not informed about the specific treatment of each animal group carried out the tests. Results were expressed as means ± S.E.M. and the analysis of variance was performed by ANOVA test. A Bonferroni’s significant difference procedure was used as a post hoc comparison. *P* values less than 0.05 were considered significant. Data were analyzed using the “Origin 9.1” software.

## 3 RESULTS

### 3.1 Effects of Ultramicronized PEA on the Onset of Morphine Tolerance in CCI Rats

**Fig. (2):**
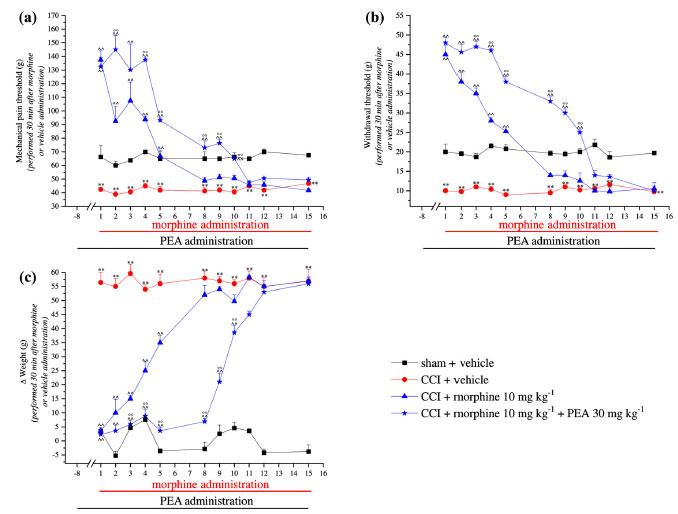
Effect of repeated administrations of PEA on the onset of morphine tolerance. PEA (30 mg kg^-1^), or vehicle (1% CMC), were administered p.o. daily (in the evening) for the duration of the experiment starting on day -8. Starting from day 1, daily acute morphine treatment (10 mg kg^-1^ s.c.) or vehicle (saline solution) were administered. The measurements of pain threshold were performed in the morning, 16 h after PEA administration and 30 min after morphine injection by (**a**) Paw pressure, (**b**) von Frey and (**c**) Incapacitance tests. Data reported in the graphs showed the results obtained 30 min after morphine or vehicle administration. Data are expressed as the mean ± S.E.M. of values from 10 rats analyzed in two different experimental sets. *P < 0.05 and **P < 0.01 *vs.* vehicle + vehicle; ^^*P* < 0.01 *vs.* CCI + vehicle; °*P* < 0.05 and °°*P* < 0.01 *vs.* CCI + morphine.

The CCI surgery determined a significant decrease in the pain threshold in comparison to the control group (Fig. **2**). Indeed, eight days after the ligation of the sciatic nerve (day 1), the weight tolerated by the animals on the ipsilateral paw decreased from about 70 g (sham + vehicle) to about 40 g (CCI + vehicle) (Fig. **2a**). The preemptive treatment with PEA (30 mg kg^-1^ since day -8) evoked an anti-hyperalgesic effect, significantly increasing the animal’s pain threshold, although values of the control group were not reached (Supplementary Fig. **S1**; CCI + PEA group, pretest). On day 1, 10 mg kg^-1^ morphine, with or without PEA, significantly increased the ipsilateral pain threshold compared to control animals (Fig. **2a**). In the following days, morphine efficacy progressively decreased and, on day 8, CCI + morphine-treated animals completely lacked the analgesic response whereas rats in the morphine + PEA group (Group D) showed a delayed onset of tolerance till day 11 (Fig. **2a**). Similar results were obtained evaluating the response of the ipsilateral paw to a non-noxious mechanical stimulus (von Frey test; Fig. **2b**). As shown in Fig. (**2c**), CCI animals showed spontaneous pain expressed as Δ weight between the contralateral and ipsilateral paws in comparison to the sham animals. Morphine injections counteracted CCI-induced hypersensitivity up to day 8, the effect being prolonged to day 11 by PEA (Fig. **2c**).

### 3.2 Effects of Different Combinations of PEA and Morphine on Morphine Analgesia in CCI Rats

**Fig. (3):**
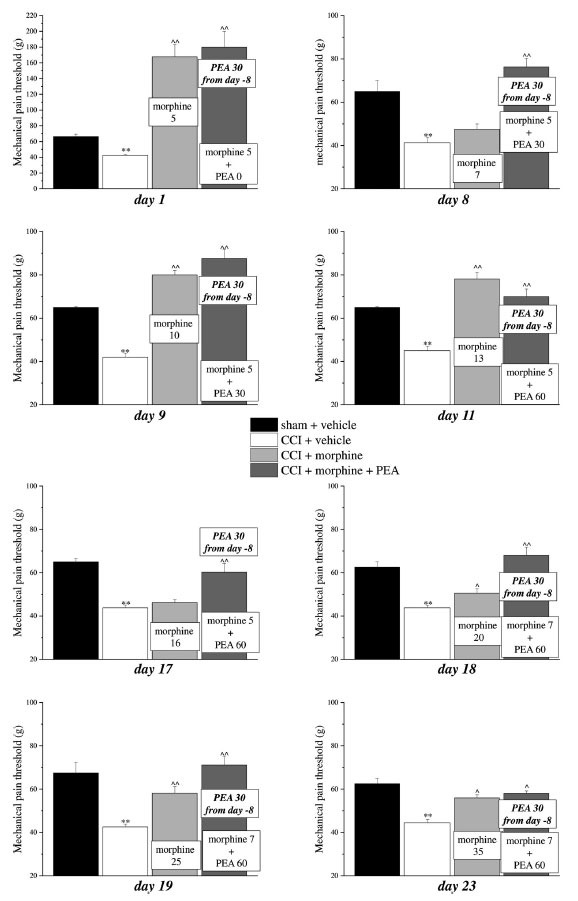
Induction of antinociception over time with different combinations of morphine and PEA by Paw pressure test. Rats were treated with vehicle or PEA (30 mg kg^-1^) p.o. daily (in the evening) for the duration of the experiment starting on day -8. To maintain a significant increase of pain threshold (90 ± 10 g) *vs.* baseline (sham + vehicle), daily beginning on day 1, increasing doses of morphine (5-35 mg kg^-1^) were injected s.c. to group CCI + vehicle. Different combinations of morphine (5-7 mg kg^-1^, s.c.) and PEA (30-60 mg kg^-1^, p.o.) were administered to the CCI + morphine + PEA group. Mechanical hyperalgesia was measured every day, in the morning, 30 min after morphine or/and PEA acute administration by Paw pressure test. Representative results obtained on days 1, 8, 9, 11, 17, 18, 19 and 23 are shown 30 min after treatments. Data are expressed as the mean ± S.E.M. of values from 10 rats analyzed in two different experimental sets. **P < 0.01 *vs.* the normal pain threshold (sham + vehicle); ^*P* < 0.05 and ^^*P* < 0.01 *vs.* CCI + vehicle.

**Fig. (4):**
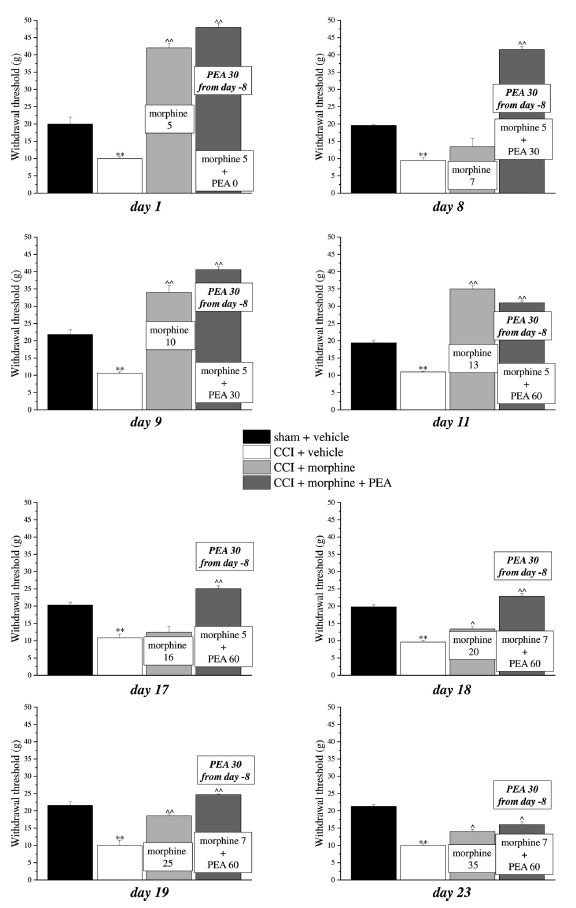
Induction of antinociception over time with different combinations of morphine and PEA by von Frey test. Rats were treated with vehicle or PEA (30 mg kg^-1^) p.o. daily (in the evening) for the duration of the experiment starting on day -8. To maintain a significant increase of pain threshold (90 ± 10 g) *vs.* baseline (sham + vehicle), daily beginning on day 1, increasing doses of morphine (5-35 mg kg^-1^) were injected s.c. to group CCI + vehicle. Different combinations of morphine (5-7 mg kg^-1^, s.c.) and PEA (30-60 mg kg^-1^, p.o.) were administered to the CCI + morphine + PEA group. Mechanical allodynia was measured every day, in the morning, 30 min after morphine or/and PEA acute administration by Paw pressure test. Representative results obtained on days 1, 8, 9, 11, 17, 18, 19 and 23 are shown 30 min after treatments. Data are expressed as the mean ± S.E.M. of values from 10 rats analyzed in two different experimental sets. **P < 0.01 *vs.* the normal pain threshold (sham + vehicle); ^*P* < 0.05 and ^^*P* < 0.01 *vs.* CCI + vehicle.

**Fig. (5):**
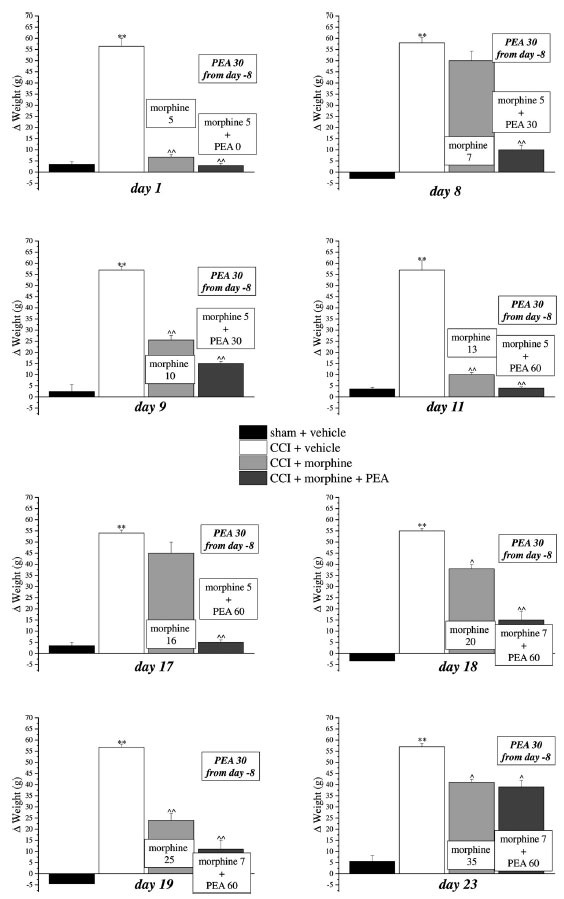
Induction of antinociception over time with different combinations of morphine and PEA by Incapacitance test. Rats were treated with vehicle or PEA (30 mg kg^-1^) p.o. daily (in the evening) for the duration of the experiment starting on day -8. To maintain a significant increase of pain threshold (90 ± 10 g) *vs.* baseline (sham + vehicle), daily beginning on day 1, increasing doses of morphine (5-35 mg kg^-1^) were injected s.c. to group CCI + vehicle. Different combinations of morphine (5-7 mg kg^-1^, s.c.) and PEA (30-60 mg kg^-1^, p.o.) were administered to the CCI + morphine + PEA group. Spontaneous pain was measured every day, in the morning, 30 min after morphine or/and PEA acute administration by Incapacitance test. Representative results obtained on days 1, 8, 9, 11, 17, 18, 19 and 23 are shown 30 min after treatments. Data are expressed as the mean ± S.E.M. of values from 10 rats analyzed in two different experimental sets. **P < 0.01 *vs.* the normal pain threshold (sham + vehicle); ^*P* < 0.05 and ^^*P* < 0.01 *vs.* CCI + vehicle.

Previous evidence showed the capacity of PEA to increase morphine analgesia when it was co-administered with the opioid in naïve rats [13, 29]. This peculiarity allows to reduce the dose of morphine utilized during a chronic treatment without penalizing the analgesic effect. We next tested this characteristic also in neuropathic animals and we decided to set up a protocol focused on maintaining a stable analgesia with the association of acute PEA and morphine in addition to the use of preemptive PEA (30 mg kg^-1^ daily in the evening from day -8). Mechanical hyperalgesia and allodynia and spontaneous pain were daily recorded by Paw pressure, von Frey and Incapacitance tests, respectively (Figs. **3-5**). The purpose was to maintain stable analgesia on the ipsilateral paw (arbitrarily fixed at 90 ± 10 g). Sham and CCI rats were treated either with vehicle or PEA (30 mg kg^-1^) from day -8 till the end of the experiment (day 23). From day 1 to day 23, daily pain measurements were performed in the morning, 16 h after the last preemptive PEA administration, before and 30 min after increasing doses of morphine (5-35 mg kg^-1^, s.c.) in CCI + morphine-treated group or a different combination of morphine (5-7 mg kg^-1^, s.c.) and PEA (30-60 mg kg^-1^, p.o.) in CCI + morphine + PEA-treated group. Figs. (**3**, **4** and **5**) show the results of representative days. On day 1, 5 mg kg^-1^ morphine was needed in both groups (CCI + morphine and CCI + morphine + PEA) to reach a value of about 180 g at the Paw pressure test (Fig. **3**). On day 9, 10 mg kg^-1^ morphine was needed to maintain the pain threshold up to 80 g whereas 5 mg kg^-1^ was enough in combination with PEA 30 mg kg^-1^ to reach the same analgesic effect. On day 11, 13 mg kg^-1^ morphine was necessary to maintain a stable analgesic effect while only morphine 5 mg kg^-1^ in association with PEA 60 mg kg^-1^ was needed in CCI + morphine + PEA treated rats. Over the days, we have come to administer morphine 16 mg kg^-1^ (day 17), 20 mg kg^-1^ (day 18), 25 mg kg^-1^ (day 19) and 35 mg kg^-1^ (day 23) in comparison to morphine 5 mg kg^-1^ plus PEA 60 mg kg^-1^ (day 17), 7 mg kg^-1^ plus PEA 60 (days 18-23) (Fig. **3**). Figs. (**4** and **5**) show similar results obtained by measuring the response of animals to a mechanical non-noxious stimulus and the spontaneous pain by the von Frey and the Incapacitance tests, respectively. The increase of morphine and PEA dosages used to evoke the same level of analgesia in the two groups (CCI + morphine and CCI + morphine + PEA) is summarized in Fig. (**6**). The columns in pink represent morphine doses used over time whereas the green columns show the doses of PEA combined with the opioid to potentiate analgesia.

### 3.3 Effects of Preemptive and Acute Administrations of PEA in CCI Rats

We also evaluated the anti-hyperalgesic effect of acute increasing doses of PEA, in addition to its preemptive treatment, in CCI rats (Supplementary Fig. **S1**). Preemptive PEA (30 mg kg^-1^ from day –8 to day 4; 60 mg kg^-1^ days 5-23) was administered daily in the evening from day -8. From day 1 till day 23, daily pain measurements were performed by the Paw pressure test in the morning, 16 h after the last preemptive PEA administration and 30 min after the acute increasing doses of PEA treatment (30-120 mg kg^-1^). PEA preemptive treatment enhanced the weight tolerated by the animals on the ipsilateral paw in comparison to the CCI + vehicle group from day 1 to the end of the experiment highlighting an anti-hypersensitivity effect. Pain assessments at 30 min after the daily acute PEA administration showed a slightly increase that reached the statistical significance on days 3, 4 and 17 (Supplementary Fig. **S1**).

### 3.4 Histological Analysis of Mast Cells in the Nervous Tissue

**Fig. (7):**
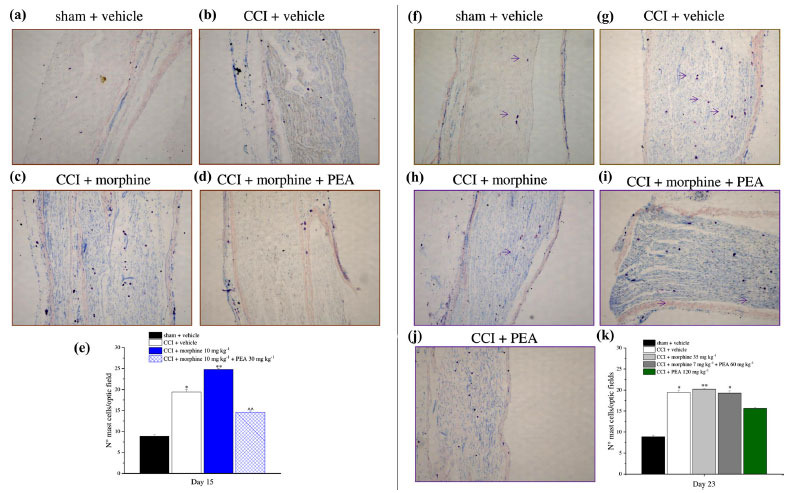
Mast cells analysis in the sciatic nerve. The number of mast cells in the sciatic nerve was studied by histochemical GIEMSA staining on days 15 or 23, on the basis of the experiments conducted. (**a-d**) Rats were treated with PEA (30 mg kg^-1^), or vehicle, p.o. daily (in the evening) for the duration of the experiment starting on day -8. On day 1, daily acute morphine treatment (10 mg kg^-1^ s.c.) started. Animals were sacrificed on day 15, when tolerance to the antinociceptive effect of morphine was developed. Representative images of the sciatic nerve at 10X magnification are shown. Quantitative analysis (**e**) of cellular density (N° mast cells/optic field) was reported. (**f-i**) Rats were treated with vehicle or PEA (30 mg kg^-1^) p.o. daily (in the evening) for the duration of the experiment starting on day -8. To maintain a significant increase of pain threshold (90 ± 10 g) *vs.* baseline (sham + vehicle), beginning on day 1, daily increasing doses of morphine (5-35 mg kg^-1^) were injected s.c. to the CCI + vehicle group. Different combinations of morphine (5-7 mg kg^-1^, s.c.) and PEA (30-60 mg kg^-1^, p.o.) were administered to the CCI + morphine + PEA group. Animals were sacrificed on day 23. Representative images of the sciatic nerve at 10X magnification are shown. (**j**) Rats were treated with PEA (30 mg kg^-1^ days -8-4; 60 mg kg^-1^ days 5-23). From day 1 till day 23, acute increasing doses of PEA treatment (30-120 mg kg^-1^) were administered in addition to the preemptive treatment. Animals were sacrificed on day 23. Representative images of sciatic nerve at 10X magnification are shown. (**k**) Quantitative analysis of cellular density (N° mast cells/optic field) was reported for treatment explained from f to j. The reported values are the means ± S.E.M. of the measurements of individual animals. At least five independent arbitrary optic fields collected from each tissue of each animal were analyzed. *P < 0.05 and **P < 0.01 *vs.* sham + vehicle; ^^*P* < 0.01*vs.* CCI + vehicle.

After the behavioral experiments, animals were sacrificed and the nervous tissue (sciatic nerve, DRGs, spinal cord and meninges from the thoracic portion of the spinal cord) was analyzed to evaluate the effect of treatments on mast cell number. Fig. (**7**) shows the number of mast cells in the sciatic nerve detected by GIEMSA-staining in all treatment protocols. The number of mast cells was significantly higher in neuropathic animals (CCI + vehicle group; Fig. **7b**) on day 15 with respect to the control group (Fig. **7a**). The same increase was observed in morphine-tolerant animals (CCI + morphine group; Fig. **7c**), while the daily treatment with PEA counteracted this phenomenon as illustrated in Fig. (**7d**). The quantitative analysis is reported in Fig. (**7e**).

Mast cell analysis was also performed on day 23 in the groups of animals treated with different combinations of morphine and PEA (Figs. **7f-7i**). An increase was found in all neuropathic animals in comparison to the sham + vehicle group regardless of the treatment group (Figs. **7h**, **7i** and **7j**, respectively). The quantitative analysis is reported in Fig. (**7k**).

**Fig. (8):**
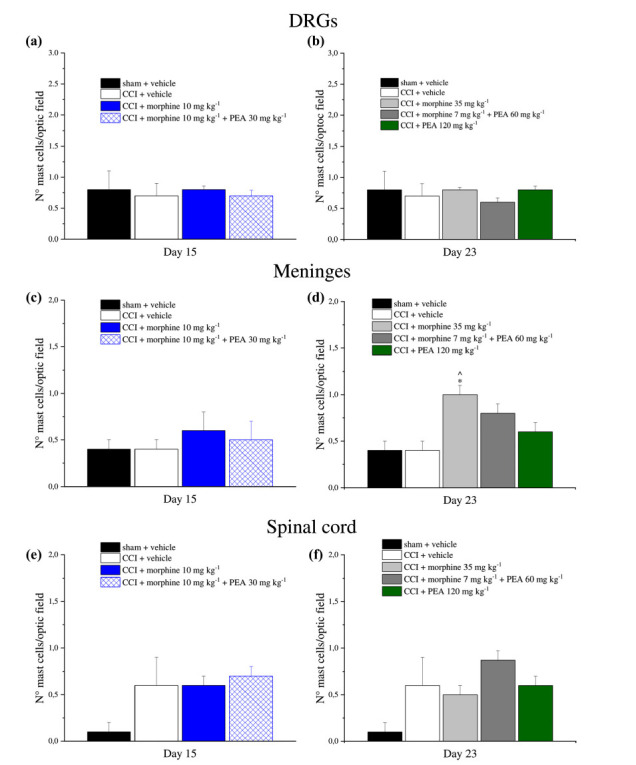
Mast cells analysis in DRGs, spinal cord meninges and spinal cord. The number of mast cells in (**a** and **b**) DRGs, (**c** and **d**) meninges (spinal cord level) and (**e** and **f**) lumbar spinal cord was studied on days 15 or 23, on the basis of the experiments conducted. Rats sacrificed on day 15 were treated with PEA (30 mg kg^-1^), or vehicle, p.o. daily (in the evening) for the duration of the experiment starting on day -8. On day 1, daily acute morphine treatment (10 mg kg^-1^ s.c.) started till the onset of tolerance. Rats sacrificed on day 23 were treated with vehicle or PEA (30 mg kg^-1^) p.o. daily (in the evening) for the duration of the experiment starting on day -8. To maintain a significant increase of pain threshold (90 ± 10 g) *vs.* baseline (sham + vehicle), beginning on day 1, daily increasing doses of morphine (5-35 mg kg^-1^) were injected s.c. to CCI + vehicle group. Different combinations of morphine (5-7 mg kg^-1^, s.c.) and PEA (30-60 mg kg^-1^, p.o.) were administered to the CCI + morphine + PEA group. Rats sacrificed on day 23 were treated with PEA (30 mg kg^-1^, days -8-4; 60 mg kg^-1^, days 5-23). From day 1 till day 23, acute increasing doses of PEA treatment (30-120 mg kg^-1^) were administered in addition to the preemptive treatment. Graphs show the quantitative analysis of cellular density (N° mast cells/optic field) reported for all the explained treatments. The reported values are the means ± S.E.M. of the measurements of individual animals. At least five independent arbitrary optic fields collected from each tissue of each animal were analyzed. *P < 0.05 *vs.* sham + vehicle; ^*P* < 0.05 *vs.* CCI + vehicle.

The number of mast cells was not altered in DRGs (Figs. **8a** and **8b**) and the spinal cord (**8e** and **8f**) either on day 15 or day 23 whereas a significant increase was detected in spinal meninges by 35 mg kg-1 morphine in comparison to sham + vehicle and CCI + vehicle groups on day 23 (Fig. **8d**). Furthermore, on day 15 no significant alteration in mast cell number was observed in all treatment groups (Fig. **8c**).

### 3.5 Histamine and N-methylhistamine Plasma Levels

**Fig. (9):**
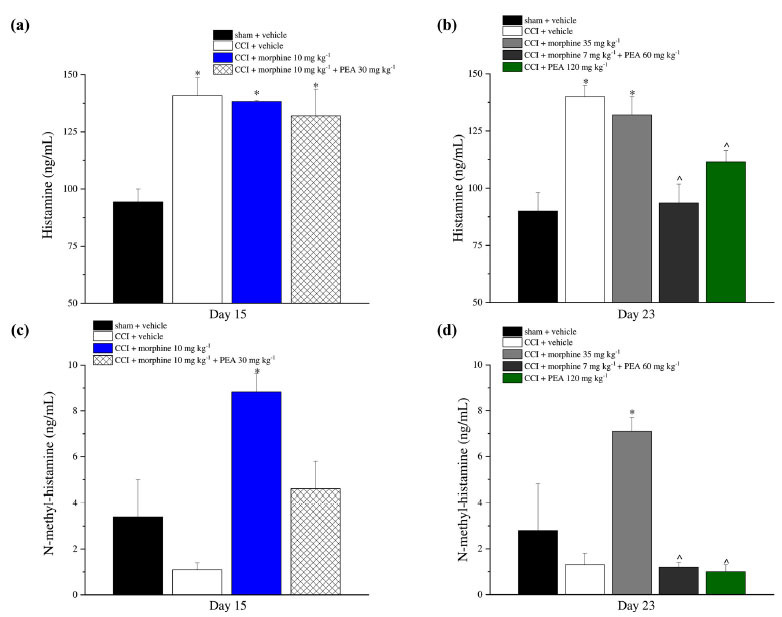
Histamine and N-methyl-histamine evaluation on plasma. Histamine (**a** and **b**) and N-methyl-histamine (**c** and **d**) were dosed on plasma on days 15 or 23 by HPLC. Rats sacrificed on day 15 were treated with PEA (30 mg kg^-1^), or vehicle, p.o. daily (in the evening) for the duration of the experiment starting on day -8. On day 1, daily acute morphine treatment (10 mg kg^-1^ s.c.) started till the onset of tolerance. Rats sacrificed on day 23 were treated with vehicle or PEA (30 mg kg^-1^) p.o. daily (in the evening) for the duration of the experiment starting on day -8. To maintain a significant increase of pain threshold (90 ± 10 g) *vs.* baseline (sham + vehicle), beginning on day 1, daily increasing doses of morphine (5-35 mg kg^-1^) were injected s.c. to the CCI + vehicle group. Different combinations of morphine (5-7 mg kg^-1^, s.c.) and PEA (30-60 mg kg^-1^, p.o.) were administered to the CCI + morphine + PEA group. Rats sacrificed on day 23 were treated with PEA (30 mg kg^-1^ days -8-4; 60 mg kg^-1^ days 5-23). From day 1 till day 23, acute increasing doses of PEA treatment (30-120 mg kg^-1^) were administered in addition to the preemptive treatment. Data are expressed as the mean ± S.E.M. of values from 10 rats analyzed in two different experimental sets. *P < 0.05 *vs.* sham + vehicle; ^*P* < 0.05 *vs.* CCI + vehicle.

Histamine was significantly increased in neuropathic animals (CCI + vehicle) and in morphine tolerant animals sacrificed on day 15 (CCI + morphine 10 mg kg^-1^) in comparison to the control group; no effects were observed in response to 30 mg kg^-1^ PEA treatment (Fig. **9a**; Group D). An increase in histamine levels was also evoked by increasing doses of morphine (Fig. **9b**; Group G). The daily co-administration of PEA 60 mg kg^-1^ and morphine 7 mg kg^-1^ reduced this trend as well as the administration of PEA alone in CCI animals (Fig. **9b**; Groups H and I).

N-methyl-histamine was significantly altered by both protocols of treatment with morphine (10 mg kg^-1^ and 35 mg kg^-1^ morphine; Figs. (**9c** and **9d**); Groups C and G, respectively). PEA 60 mg kg^-1^ and PEA 120 mg kg^-1^ (Groups H and I) reduced N-methylhistamine plasma levels (Fig. **9d**) whereas PEA 30 mg kg^-1^ was ineffective (Fig. **9c**; Group D).

### 3.6 Glia Analysis

**Fig. (10):**
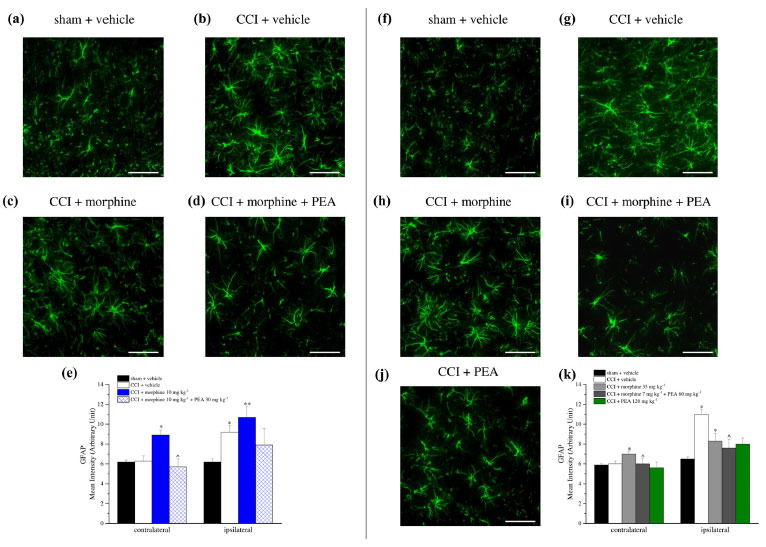
Astrocyte analysis in the dorsal horn of the lumbar portion of the spinal cord on both contralateral and ipsilateral sides. Representative images of anti-GFAP immunohistochemistry at 20X magnification are reported for rats sacrificed on day 15 (**a-d**) and on day 23 (**f-j**). Quantitative analysis was reported as an arbitrary unit of GFAP mean intensity (figures **e**, **k**) and data were expressed as mean ± S.E.M. of 3 different fields of 3 specimens for each of 6 samples from 6 different animals. *P < 0.05 and **P < 0.01 *vs.* sham + vehicle; ^*P* < 0.05 *vs.* CCI + vehicle.

The lumbar spinal cord was analyzed to detect astrocytes and microglia changes in response to different treatments (Fig. **10a-k**). CCI significantly increased the GFAP-fluorescence intensity in the dorsal horn of the spinal cord of the ipsilateral side (Fig. **10e**). Ten mg kg^-1^ and increasing doses of morphine significantly enhanced the astrocyte's density not only on the ipsilateral side but also on the contralateral one (Fig. **10e** and **10k**). PEA 30 mg kg^-1^ reduced GFAP expression on the contralateral side of tolerant animals (CCI + morphine + PEA group) (Fig. **10e**) whereas PEA administered at increasing doses was significantly effective on both sides (CCI + morphine increasing doses + PEA group) (Fig. **10k**).

**Fig. (11):**
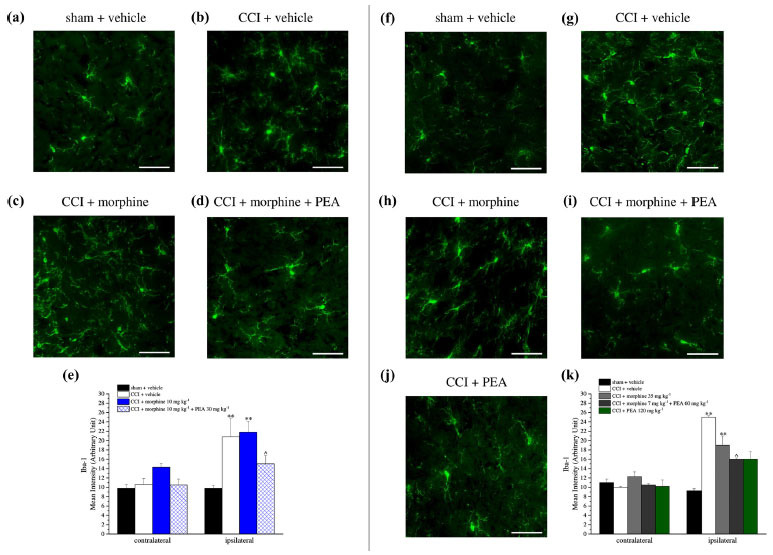
Microglia analysis in the dorsal horn of the lumbar portion of the spinal cord on both contralateral and ipsilateral sides. Representative images of anti-Iba-1 immunohistochemistry at 20X magnification are reported for rats sacrificed on day 15 (**a-d**) and on day 23 (**f-j**). Quantitative analysis was reported as an arbitrary unit of Iba-1 mean intensity (figures **e**, **k**) and data were expressed as mean ± S.E.M. of 3 different fields of 3 specimens for each of 6 samples from 6 different animals. **P < 0.01 *vs.* sham + vehicle; ^*P* < 0.05 *vs.* CCI + vehicle.

Microglia was analyzed by Iba-1 immunoreactivity (Fig. **11a-k**). Iba-1 staining was significantly increased on the ipsilateral side of CCI + vehicle, CCI + morphine 10 mg kg^-1^ and CCI + morphine increasing doses treated animals. This increase was reduced by PEA treatment in all groups (Figs. **11e** and **11k**).

## 4 DISCUSSION

An American survey based on 3575 responders found a prevalence rate of neuropathic pain of 9.8% [30]. Data from a more recent and larger survey (*i.e*., 24,925 respondents) estimated the prevalence rate of probable neuropathic pain among USA people to be 10% [31]. Based on these and other available data it follows that neuropathic pain represents a relevant clinical need. Although IASP considers indispensable the use of opioids for the treatment of severe short-lived pain during acute painful events as well as at the end of life in cancer patients, the role of opioids in the treatment of chronic pain raises serious concerns and caution is recommended when opioids are prescribed in this setting [1]. Thus, due to the availability of safer drugs, the use of strong opioids in the treatment of chronic neuropathic pain is only recommended for the advanced lines of treatment (with the exception of tramadol as potential second line treatment) [5, 32]. Agents able to modulate the effects of opioids may represent a relevant advantage in the management of neuropathic pain. To this purpose, PEA represents a very promising candidate. Various mechanisms contribute to the anti-inflammatory and analgesic effects of PEA. Non-neuronal cells are considered the main cellular targets of PEA [14], with PEA being able to exert an autacoid local injury antagonism (ALIA mechanism) in order to down-modulate hyper-releasability of pro-inflammatory and sensitizing mediators [9, 33, 34]. The ALIA mechanism is considered to involve both direct and indirect agonism at different receptors belonging to the so-called endocannabinoidome, *i.e*., the extended endocannabinoid system [35]. In fact, PEA is a direct agonist of the nuclear peroxisome proliferator-activated receptor-alpha (PPAR-α) [36] and the orphan receptor G-protein coupling, GPR55 [37]. Moreover, PEA indirectly activates other endocannabinoid receptors through the so-called “entourage” effect, that is the ability to (i) increase the synthesis of endocannabinoid mediators, (ii) decrease their degradative pathways or (iii) improve their binding affinity to the respective receptors [38-41]. Thus, the exogenous administration of PEA-provided bioavailable formulations are used-may contribute to counteract pathological conditions (*e.g*., neuroinflammation) in which low levels of endogenous PEA prevent its protective activity [18, 42]. In this study, we dealt with the characterization of the effects of PEA according to a study design based on a single agent PEA pre-treatment followed by the co-administration of PEA and morphine in a neuropathic pain rat model, *i.e*., the CCI, that is considered similar to human features of neuropathic pain [21], including the occurrence of allodynia [21, 43]. Based on our previous experience [13], the study design was rationally planned to maintain a stable pain-relieving effect on the ipsilateral paw (arbitrarily fixed 90 ± 10 g) through the administration of preemptive PEA followed by the concomitant administration of PEA and morphine (*i.e*., daily injections at the lowest effective dose). In fact, as previously mentioned, since reduced levels of endogenous PEA have been observed mainly in the spinal cord after 3 days, but also at the dorsal raphe and rostral ventral medulla after 7 days from CCI of the sciatic nerve [18], the preemptive PEA administration may relevantly contribute in the maintaining of adequate PEA levels. We have previously shown that PEA administered p.o. [13] was able to increase the acute antinociceptive efficacy of morphine. Furthermore, PEA prolonged the response to morphine by delaying the onset of morphine tolerance [10, 13]. Interestingly, in the present study, we were able to extend these data in the CCI neuropathic pain model in the rat, through a set of pain sensitivity behavioral experiments. In animals supplemented with PEA, morphine doses needed to obtain an anti-hyperalgesic and an anti-allodynic effect were significantly lower compared to animals treated with morphine alone. In particular, during the first week, rats treated with the drugs combination showed a higher pain threshold compared to rats treated with morphine alone, despite the 30% lower dose of morphine used in the combination treatment group (day 8). Although in the subsequent days, the pain threshold decreased in both groups, the decrease was more pronounced in the morphine alone group. The ability of PEA to modulate the onset of morphine tolerance was clearly shown by comparing data obtained by the Paw pressure test between CCI + morphine 10 mg kg^-1^ group and CCI + morphine 10 mg kg^-1^ + PEA 30 mg kg^-1^ group. In the former group, in fact, tolerance developed 3 days earlier than in the second group. Several factors contribute to the development of morphine tolerance. Ion channels, receptors, cells, and other biological components are involved in the mechanisms underlying this phenomenon, including receptor desensitization, phosphorylation, neuroinflammation and microglial activation by opioids [44-49]. The dysregulation of microglia functions is in fact not only involved in psychiatric or neurodegenerative diseases [50, 51], but also in the induction of morphine tolerance, either in its initiation and maintenance [52, 53]. Also, there is evidence that morphine and in general opioids, can affect several functions of immune cells [54]. In particular, morphine may exert its proinflammatory activity through the involvement of the TLR4 signaling cascade that regulates the expression of several inflammation molecules [55-57]. The activated microglia secrete proinflammatory cytokines that contribute to increase the hyperactivity of dorsal horn neurons, induce central sensitization, and reduce the morphine antinociception [58, 59]. In addition, a crosstalk between microglia and astrocytes has been assessed, they both respond to neuronal injury through changes that alter CNS tissues [60]. In a previous study, our group confirmed that chronic administration of morphine indeed activates microglia and astrocytes in the dorsal horn of the lumbar spinal cord [19]. Thus, in the present study, we evaluated microglia and astrocyte activation in lumbar spinal cord of treated and untreated rats by studying Iba-1 and GFAP biomarker expression, respectively. Overall, GFAP-fluorescence intensity was increased in the dorsal horn of the spinal cord of the ipsilateral side of CCI untreated animals as well as in both sides of animals treated with morphine 10 mg kg^-1^, thus confirming the ability of a chronic morphine treatment to determine astrocyte activation. Similar findings were reported for Iba-1 expression. Iba-1 staining was significantly increased on the ipsilateral side of controls (CCI + vehicle) as well as of treated animals (CCI + morphine 10 mg kg^-1^; CCI + morphine increasing doses). The oral administration of PEA, according to the different schedules used, was able to reduce both GFAP and Iba-1 immunoreactivity. Thus, we were able to confirm relationships between the activation of microglia and astrocyte morphine tolerance as well as to show the protective effects of PEA in this context in a CCI rat model. Since as above mentioned, microglia and astrocytes respond to pro-inflammatory signals deriving from several cells, including immune cells [54], due to the role that mast cells play in the mechanism of action of PEA, we characterized mast cells in terms of numbers and degranulation. In particular, the number of mast cells, mainly at the local level (*i.e*. injured sciatic nerve), was significantly reduced in animals treated with PEA. This was observed when PEA and morphine were co-administered at a fixed dose (morphine 10 mg kg^-1^ and PEA 30 mg kg^-1^) as well as when both were co-administered at increasing doses or when PEA was administered as a single agent at a high dose (120 mg kg^-1^) in comparison with the number of mast cells reported in case of morphine single agent treatment (*i.e*. morphine 10 mg kg^-1^ or 35 mg kg^-1^). Very low levels of mast cells were instead observed in the other study tissues (*i.e*. DRGs, meninges, lumbar spinal cord). These data are in agreement with those that suggest the negative control of mast cell activity as one of the main cellular mechanisms by which PEA exerts its *in vivo* protective activities including those against neurogenic inflammation and neuropathic pain [61-64]. However, more information is needed to understand how PEA modulates mast cell degranulation and how this may impact on glial cells. Recent *in vitro* evidence shows that PEA counteracts mast cell degranulation induced by substance P in RBL-2H3 cells by enhancing the synthesis of the endocannabinoid 2-arachidonoylglycerol (2-AG) [65]. Interestingly, PEA was able to reduce substance P-induced release of one of the main mast cell mediators, *i.e*., histamine [65]. Overall, it is well recognized that the release of histamine by mast cells in response to nerve injury or damage contributes to pain hypersensitivity [66-68]. The decrease of mast cell degranulation, here evaluated in terms of reduced plasma levels of histamine and its N-methyl-histamine metabolite, was mainly observed at intermediate-high doses of PEA (with or without morphine) whereas the lowest dose (*i.e*., 30 mg kg^-1^) was not able to reduce plasma histamine and N-methyl-histamine levels compared with the controls. Similarly, the plasma levels of the metabolite N-methyl-histamine were significantly increased in animals treated with morphine alone and decreased in the animals treated with the drug combination at the highest PEA dose (*i.e*. 60 mg kg^-1^) or with PEA alone (*i.e*. 120 mg kg^-1^) compared with controls. Although no data are available on the reduction of histamine plasma levels following PEA treatment in *in vivo* neuropathic pain models, there is evidence of reduced levels of histamine by PEA in *ex vivo* canine skin models [69, 70]. In particular, ultramicronized PEA significantly decreased the release of histamine from immunologically challenged canine skin mast cells and canine skin organ cultures stimulated with the secretagogue compound 48/80 [69, 70].

## CONCLUSION

Overall, we showed that in the CCI neuropathic pain model the administration of the natural bioactive compound PEA according to the study design fully satisfied the endpoints of this study. In particular, the observed pain-relieving effects of PEA may be mainly due to the preemptive administration of PEA, useful as mentioned, to restore the endogenous levels of PEA before the opioid treatment. PEA is per se a compound available in some countries and referred to as “Food for Special Medical Purposes”. Micronized or ultramicronized PEA formulations have been investigated in several clinical conditions including chronic pain [71], migraine [72], and neuropathic pain [73, 74]. Overall, in these pathologies, PEA has been administered according to different schedules (*i.e*. 600 mg/die [72, 73]; 600 mg x 2/daily [74]) showing promising results. To summarize, based on our results, a clinical trial investigating PEA as a preemptive treatment and then in combination with opioids could be implemented and it could importantly contribute to improve their modulation in the treatment of chronic neuropathic pain.

## Figures and Tables

**Fig. (1):**
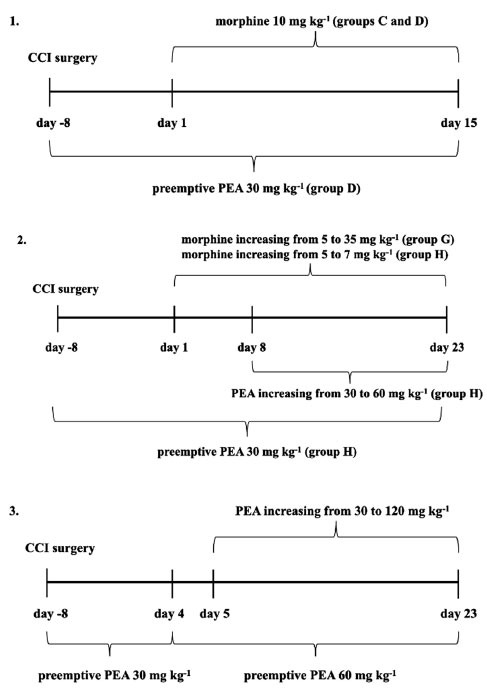
Design of the study. The dose of morphine and PEA varied according to the experiment. Experiments were conducted and set up to evaluate if PEA: 1) delays the onset of morphine tolerance in CCI rats; 2) potentiates morphine analgesia in CCI rats and 3) reduces CCI-induced hyperalgesia.

**Fig. (6):**
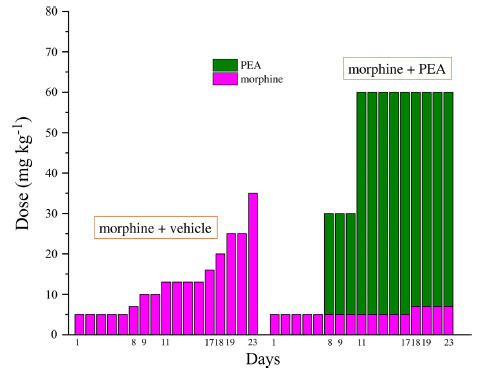
Comparison among equi-analgesic dosages of morphine/PEA combinations and morphine. Rats were treated with vehicle or PEA (30 mg kg^-1^) p.o. daily (in the evening) for the duration of the experiment starting on day -8. To maintain a significant increase of pain threshold (90 ± 10 g) *vs.* baseline (sham + vehicle), daily beginning on day 1, increasing doses of morphine (5-35 mg kg^-1^) were injected s.c. to group CCI + vehicle. Different combinations of morphine (5-7 mg kg^-1^, s.c.) and PEA (30-60 mg kg^-1^, p.o.) were administered to the CCI + morphine + PEA group. The dosages in mg kg^-1^ necessary to maintain the required anti-nociceptive effect every day (Paw pressure test; days 1-23) are reported. Measurements were performed in the morning, 30 min after morphine or/and PEA acute administration. Each group consists of 10 rats analyzed in two different experimental sets.

## Data Availability

The data presented in this study are available on request from the corresponding author.

## LIST OF ABBREVIATIONS

CCI: Chronic Constriction Injury
CMC: Carboxymethylcellulose
2D-HPLC: Two Dimensional High Performance Liquid Chromatography
NSAIDs: Nonsteroidal Anti-Inflammatory Drugs
OCT: Optimal Cutting Temperature
PBS: Phosphate-Buffered Saline
PFA: Paraformaldehyde
PPAR-α: Peroxisome Proliferator-Activated Receptor-Alpha
QqQ: Quadrupole Mass Spectrometer
