# Supplementary material for: Ultramicronized N-palmitoylethanolamine Contributes to Morphine Efficacy Against Neuropathic Pain: Implication of Mast Cells and Glia
Source: Curr Neuropharmacol. 2023 Apr 27;22(1):88–106. doi: 10.2174/1570159X21666221128091453 (PMC10716887; doi:10.2174/1570159X21666221128091453)
Supplement: Supplementary file 1 [file CN-22-88_SD1.pdf]

Supplementary Material

Ultramicronized *N*-palmitoylethanolamine Contributes to Morphine Efficacy Against Neuropathic Pain: Implication of Mast Cells and Glia

Laura Micheli[1,#], Elena Lucarini[1,#], Stefania Nobili[1,*], Gianluca Bartolucci[2], Marco Pallecchi[2], Alessandra Toti[1], Valentina Ferrara[1], Clara Ciampi[1], Carla Ghelardini[1] and Lorenzo Di Cesare Mannelli[1]

[1]*Department of Neuroscience, Psychology, Drug Research and Child Health – NEUROFARBA –Pharmacology and Toxicology Section, University of Florence, Florence, Italy;* [2]*Department of Neuroscience, Psychology, Drug Research and Child Health – NEUROFARBA – Pharmaceutical and Nutraceutical Sciences Section, University of Florence, Florence, 50019, Italy*

1875-6190/24 $65.00+.00 **© 2024 Bentham Science Publishers**

S1: Histamine and N methyl-histamine dosage determination in rat plasma samples

The proposed isotopic dilution 2D-HPLC-MS/MS method to quantify the analytes in rat plasma samples was carried out by using a triple quadrupole mass spectrometer (QqQ) operating in multi-reaction monitoring (MRM). The method parameters were reported stepwise as follows:

1. the solvents for the HPLC pumps were: ultrapure water:CH_3CN 9:1 solution added with 5mM HCOOH and 15mM $HCOONH_4$ (Solvent A), ultrapure water:CH3CN 1:9 solution added 15mM HCOOH and 5mM $HCOONH_4$ (Solvent B), Solvent C (used for sample loading): ultrapure water:CH3CN 1:9 solution added with 17,5mM HCOOH and 2,5mM $HCOONH_4$;
2. the Solvent C was used to transfer the injected sample volume from the injector to the loading column by the loading pump in isocratic mode at constant flow of 0.5 mL min^{-1};
3. the analytical column used for the histamine assay was the SeQuant® ZIC-HILIC 50x2.1mm, 3.5µm, 100Å and the loading column (or SPE cartridge) was the SeQuant® ZIC-HILIC Guard 20x2.1mm;
4. after the transfer of the sample into the loading column (loading time=2 min.), the connected valve switches in inject position, allowing the elution in counter-current of the sample by the analytical pumps system and deposition of the injected sample in the top of analytical column (Figure SF1);

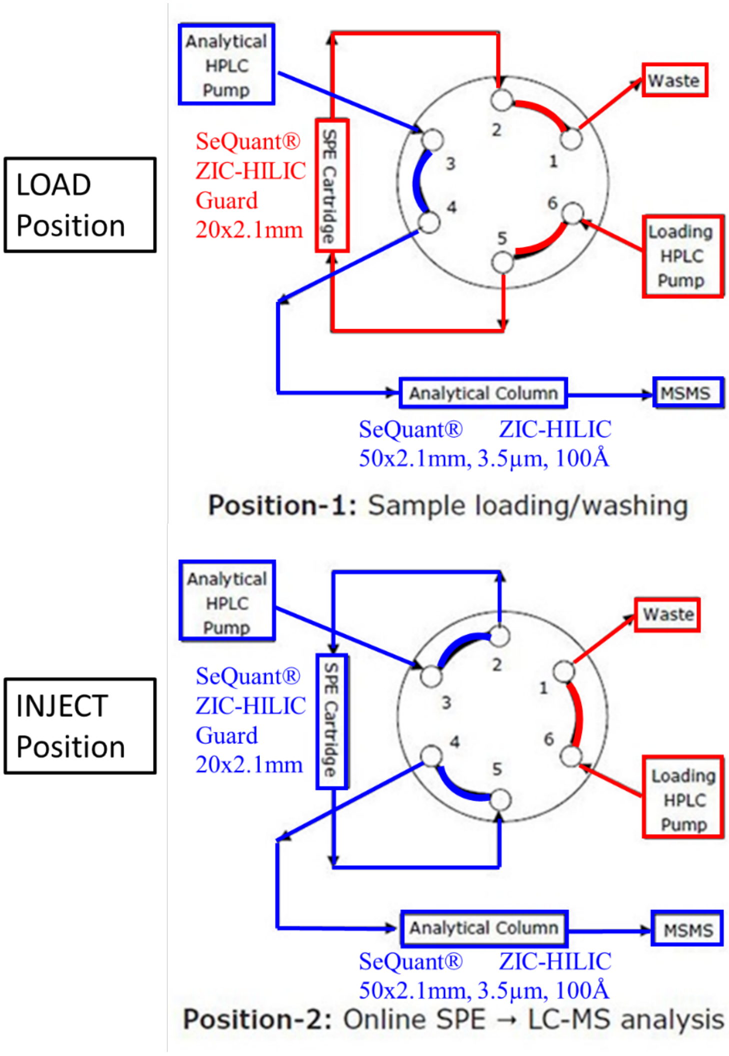

SF1: 2D-HPLC scheme of sample elution according to the switching valve position

5. The gradient elution program of analytical pumps system was developed as follows:

Time (min)	A%	B%	Flow (mL/min)	Valve Position
0,00	10	90.0	0.25	Load
2.00	10	90.0	0.25	Inject
9.00	80	20	0.25	
12.00	80	20	0.25	
12.01	10	90	0.25	Load
25.00	10	90	0.25	

The internal standard (IS) and analytes were monitored by Multiple Reaction Monitoring (MRM) mode following the most significant and abundant fragment ions. The MRM parameters were reported in table ST1.

ST1: MRM parameter used to monitor the internal standard and analytes in the 2D-HPLC-MS/MS proposed method.

Name	Precursor Ion (m/z)	Quantifier Ion (m/z)	Qualifier Ion (m/z)
Histamine-d4 (IS)	116.0	99.0	-
Histamine	112.0	95.0	68.0
N-methyl-histamine	126.0	109.0	81,0

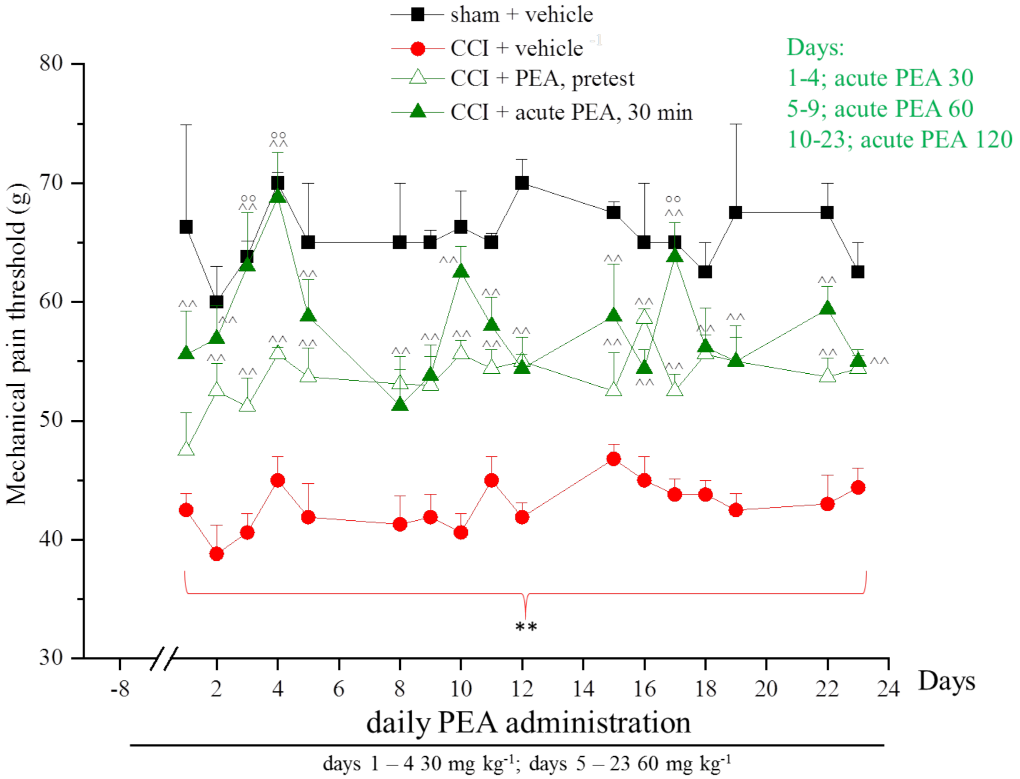

Supplementary Fig. (S1). Rats were treated with PEA (30 mg kg^{-1} days -8-4; 60 mg kg^{-1} days 5-23). From day 1 till day 23, acute increasing doses of PEA treatment (30-120 mg kg^{-1}) were administered in addition to the preemptive treatment. Mechanical hyperalgesia was assessed 16 h after preemptive PEA administration and 30 min after the acute daily injection by the Paw pressure test. Data are expressed as the mean ± S.E.M. of values from 10 rats analyzed in 2 different experimental sets. ***P* < 0.01 *vs.* vehicle + vehicle, 30 min; ^^*P* < 0.01 *vs*. CCI + vehicle, 30 min; °°*P* < 0.01 *vs*. CCI + PEA pretest.
